# A protocol for analyzing the protein terminome of human cancer cell line culture supernatants

**DOI:** 10.1016/j.xpro.2021.100682

**Published:** 2021-07-22

**Authors:** Kazuya Tsumagari, Chih-Hsiang Chang, Yasushi Ishihama

**Affiliations:** 1 Department of Molecular and Cellular BioAnalysis, Graduate School of Pharmaceutical Sciences, Kyoto University, Kyoto 606-8501, Japan; 2 Center for Integrated Medical Research, Keio University School of Medicine, Tokyo 160-8582, Japan; 3 Laboratory of Clinical and Analytical Chemistry, National Institute of Biomedical Innovation, Health and Nutrition, Ibaraki, Osaka 567-0085, Japan

**Keywords:** Cell Membrane, Protein Biochemistry, Proteomics, Mass Spectrometry, Systems biology

## Abstract

Characterization of protein termini is essential for understanding how the proteome is generated through biological processes such as post-translational proteolytic events. Here, we introduce a practical protocol for terminomics to achieve simple and sensitive N- and C-terminal peptide enrichment. We apply it to the terminome analysis of culture supernatants of a human cancer cell line for the purpose of identifying ectodomain shedding substrate cleavage sites with 10 μg protein per sample.

For complete details on the use and execution of this protocol, please refer to Tsumagari et al. (2021).

## Highlights

Simple and sensitive protocol for terminomics of culture supernatants
Enrichment for both protein N- and C-terminal peptides
Profiles of cleavage sites generated by ectodomain shedding

## Graphical abstract

**Figure:**
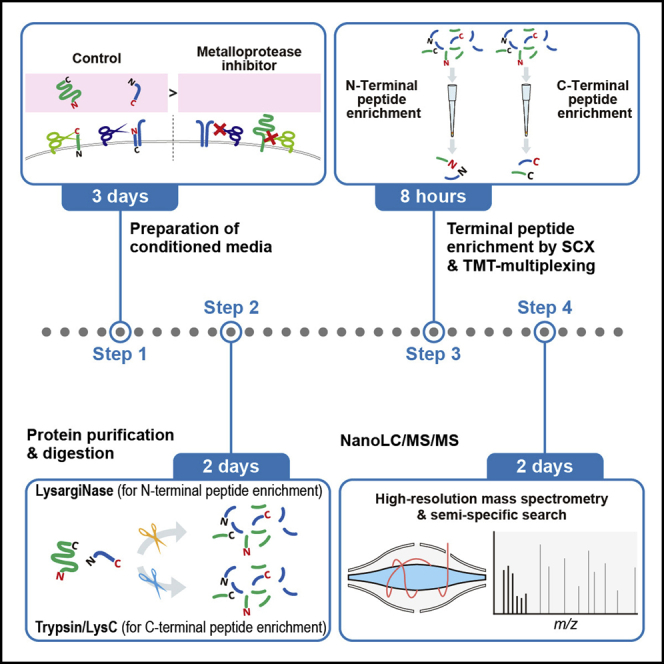

## Before you begin

Analyzing protein termini on a proteome scale, called terminomics, is a powerful approach to characterize the proteoform through defining both the N- and C-termini. Protein shedding can be transiently upregulated by certain agonists, such as phorbol 12-myristate 13-acetate (PMA), via activation of metalloproteases which have emerged as the major sheddase family (Huovila et al., 2005). To achieve large-scale identification of ectodomain shedding sites cleaved by members of the metalloprotease family, a quantitative terminomics workflow consisting of shedding activation with PMA, broad-spectrum metalloprotease inhibitor (BB-94; batimastat) treatment, SCX-based terminal peptide enrichment, TMT labeling and nano-scale liquid chromatography-tandem mass spectrometry (nanoLC/MS/MS) measurement is employed. This protocol is applicable to any cultured cells that can be maintained in serum-free medium during medium conditioning, and we have applied it to a panel of ten human cancer cell lines (Tsumagari et al., 2021). We have also used this protocol for lysates and culture supernatants of other human cell lines such as HEK293T. The protocol below describes the specific steps for the culture supernatant of A431 cells, a human cell line derived from epidermoid carcinoma, selected as a typical adherent mammalian cell line.

**Figure 1:**
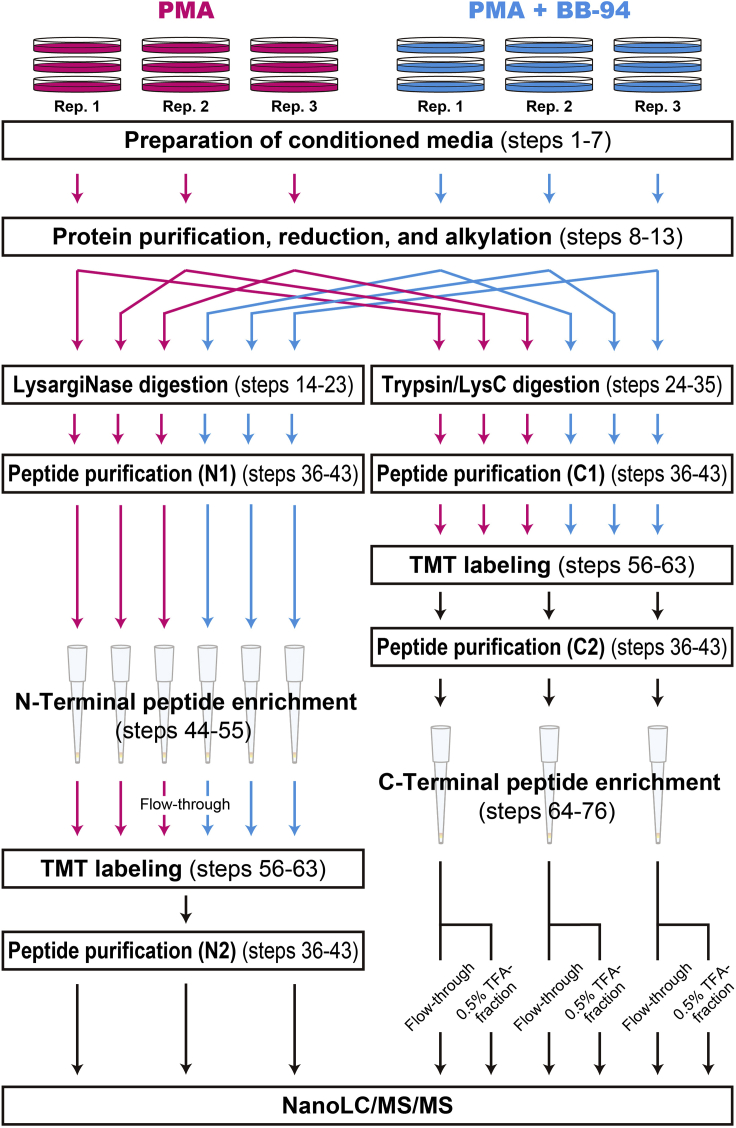
Schematic workflow Conditioned media from three 15 cm dishes are pooled to make a replicate. For N-terminomics, samples are digested with LysargiNase, subjected to N-terminal peptide enrichment, labeled with TMT, and then mixed. The multiplexed sample is divided into three parts and analyzed by triplicate LC/MS/MS runs. Note that TMT labeling should be performed after enrichment of N-terminal peptides, since the TMT label affects the enrichment efficiency. For C-terminomics, samples are digested with trypsin and LysC, labeled with TMT and mixed, divided into three parts and subjected to C-terminal peptide enrichment using three SCX-StageTips. Analyzing two fractions, the flow-through and the 0.5% TFA-eluted fraction, allows identification and quantification of C-terminal peptides on a comparable scale to the N-terminal peptide counterpart with the same amount of loaded peptides. The peptide purification process (steps 36–43) appears four times in this workflow (N1, N2, C1, and C2).

Follow the workflow shown in Figure 1 for sample preparation. In the workflow of N-terminomics (left side of Figure 1), N-terminal peptides are enriched followed by TMT labeling, while in the workflow of C-terminomics (right side of Figure 1), C-terminal peptides are enriched after TMT-labeling. This difference is due to the difference of charge distribution between LysargiNase digests (used for N-terminal peptide enrichment) and trypsin/LysC digests (used for C-terminal peptide enrichment), which affects the terminal peptide enrichment efficiency of SCX.

## Key resources table

**Table:** 

REAGENT or RESOURCE	SOURCE	IDENTIFIER
**Critical commercial assays**		

Bicinchoninic acid (BCA) assay kit	Thermo Fisher Scientific	Cat#23227
Amicon® Ultra filter (3,000 NMWL)	Millipore	Cat#UFC900396
Empore SDB-XC membrane^1^	GL Sciences	Cat#5010-30016
Empore cation-SR membrane^1^	GL Sciences	Cat#5010-30031

**Chemicals, peptides, and recombinant proteins**		

Sodium deoxycholate (SDC)	FUJIFILM Wako	Cat#194-08311
Sodium *N*-lauroylsarcosinate (SLS)	FUJIFILM Wako	Cat#192-10382
Trifluoroacetic acid (TFA)	FUJIFILM Wako	Cat#204-02743
Ammonium acetate	FUJIFILM Wako	Cat#015-02837
Dimethyl sulfoxide (DMSO)	FUJIFILM Wako	Cat#049-07213
BB-94 (batimastat)	Selleck	Cat#S7155; CAS No. 130370-60-4
Phorbol 12-myristate 13-acetate (PMA)	FUJIFILM Wako	Cat#162-23591; CAS No. 16561-29-8
Formic acid (FA)	FUJIFILM Wako	Cat#066-00461
TMT 10-plex^2^	Thermo Fisher Scientific	Cat#90406
Phenylmethylsulfonyl fluoride (PMSF)	Tokyo Chemical Industry	Cat#B3473; CAS No. 329-98-6
100 x Protease inhibitor cocktail	Sigma-Aldrich	Cat#P8340
Ethylenediamine-*N*,*N*,*N′*,*N′*-tetraacetic acid, disodium salt, dihydrate (EDTA)	Dojindo	Cat#343-01861
*O*,*O′*-Bis(2-aminoethyl)ethyleneglycol-*N*,*N*,*N′*,*N′*-tetraacetic acid (EGTA)	Dojindo	Cat#346-01312
Hydroxyamine solution (wt. 50%)	Sigma-Aldrich	Cat#438227-50ML
Tris-(2-carboxyethyl)phosphine, hydrochloride (TCEP)	Thermo Fisher Scientific	Cat#20490
2-Chloroacetamide	FUJIFILM Wako	Cat#032-09762
Trypsin	Promega	Cat#V5111
LysC	FUJIFILM Wako	Cat#129-02541
LysargiNase	Millipore	Cat#EMS0008

**Experimental models: Cell lines**		

A431	RIKEN BRC Cell Bank	Cat#RCB0202

**Software and algorithms**		

MaxQuant v.1.6.7.0	Cox and Mann, 2008	https://www.maxquant.org/
Perseus v.1.6.14.0	Tyanova et al., 2016b	https://maxquant.net/perseus/

**Deposited data**		

LC/MS/MS raw data	Tsumagari et al., 2021	PXD021378 (JPST000632)

**Other**		

Centrifuge (for 96-well plates, 50 mL-conical tubes, and Amicon Ultra centrifugal filters)	Eppendorf	Cat#5804R
Centrifuge (for microtubes)	Eppendorf	Cat#5424R

^**1**^***Alternatives:*** Commercial StageTips with SDB-XC (Cat# 7820-11200, GL Sciences) or cation-SR (Cat# 7510-11203, GL Sciences) are available.

^**2**^***Alternatives:*** TMT 6-plex or 11-plex reagent set (Cat#90061, Cat#90062, Cat#A34808) or iTRAQ reagent set (Cat#4381663, SCIEX) can be used instead of TMT 10-plex.

## Materials and equipment

### StageTip

**Figure 2:**
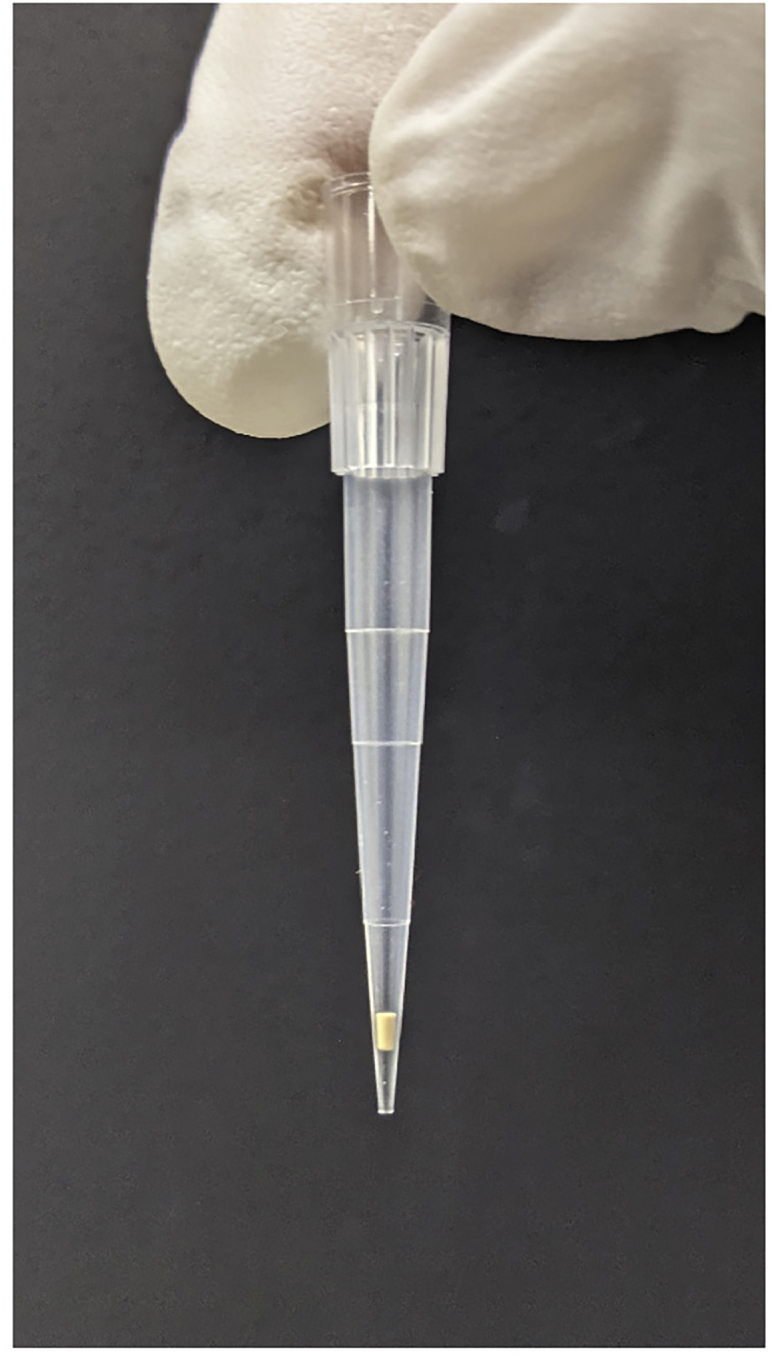
Example of StageTip An ordinary 200 μL-pipette tip with inserted double SCX membranes. The membranes are hollowed out with a 16-gauge blunt-end needle.

For peptide mixture purification (desalting) and terminal peptide enrichment, lab-made stop-and-go-extraction tips (StageTips) are used (Rappsilber et al., 2007). StageTips are ordinary pipette tips packed with small membrane incorporating chromatographic sorbents (Figure 2). In this protocol, 200-μL pipette tips packed with double 16-gauge membranes are used. The preparation of StageTips has been described in detail previously (Rappsilber et al., 2007). Nine SCX-StageTips and Sixteen SDB-StageTips in total should be prepared in advance. Alternatively, commercial StageTips are also available (described in the key resources table as alternatives to SDB-XC membrane and cation-SR membrane). For loading solutions onto the membranes, centrifugation is used with the adapter Cat#5010-21514 (GL Sciences) for microtubes or Cat#5010-21341 (GL Sciences) for 96-well plates. The loading speed of the solution through the membrane is kept at approximately 10 μL/min.

**Table:** Phase-transfer surfactant (PTS) buffer

Reagent	Final concentration	Amount
SDC (120 mM)	12 mM	0.1 mL
SLS (120 mM)	12 mM	0.1 mL
Tris-HCl, pH 8.5 (1 M)	100 mM	0.1 mL
Ultrapure water	n/a	0.7 mL
**Total**	**n/a**	**1 mL**

Mix the stock solutions just before use. The stock solutions can be stored at 25°C for three months.

**Table:** Buffer A

Reagent	Final concentration	Amount
TFA	0.1% (v/v)	0.05 mL
Acetonitrile	4% (v/v)	2 mL
Ultrapure water	n/a	47.95 mL
**Total**	**n/a**	**50 mL**

The solution is prepared just before use.

**Table:** Buffer B

Reagent	Final concentration	Amount
TFA	0.1% (v/v)	0.05 mL
Acetonitrile	80% (v/v)	40 mL
Ultrapure water	n/a	9.95 mL
**Total**	**n/a**	**50 mL**

The solution is prepared just before use.

**Table:** N-Loading buffer

Reagent	Final concentration	Amount
FA	2.5% (v/v)	0.125 mL
Acetonitrile	30% (v/v)	1.5 mL
Ultrapure water	n/a	3.375 mL
**Total**	**n/a**	**5 mL**

The solution is prepared just before use.

**Table:** C-Loading buffer

Reagent	Final concentration	Amount
TFA (10%)	0.15% (v/v)	0.075 mL
Acetonitrile	30% (v/v)	1.5 mL
Ultrapure water	n/a	3.425 mL
**Total**	**n/a**	**5 mL**

The solution is prepared just before use.

**Table:** C-0.5% TFA buffer

Reagent	Final concentration	Amount
TFA (10%)	0.5% (v/v)	0.25 mL
Acetonitrile	30% (v/v)	1.5 mL
Ultrapure water	n/a	3.25 mL
**Total**	**n/a**	**5 mL**

The solution is prepared just before use.

**Table:** SCX-activation buffer

Reagent	Final concentration	Amount
Ammonium acetate (2 M)	500 mM	1.25 mL
Acetonitrile	30% (v/v)	1.5 mL
Ultrapure water	n/a	2.25 mL
**Total**	**n/a**	**5 mL**

The solution is prepared just before use.

**Table:** Other solutions

Reagent	Note
HEPES-NaOH, pH 8.5 (200 mM)	Adjust the pH to 8.5 with NaOH. The solution can be stored at 4°C for two weeks.
BB-94 (20 mM)	Dissolved in DMSO. The solution can be stored at –20°C for three months.
PMA (5 mM)	Dissolved in DMSO. The solution can be stored at –20°C for three months.
EDTA-NaOH, pH 8.0 (200 mM)	Adjust the pH to 8.0 with NaOH. The solution can be stored at 25°C for three months.
EGTA-NaOH, pH 8.0 (200 mM)	Adjust the pH to 8.0 with NaOH. The solution can be stored at 25°C for three months.
PMSF (100 mM)	Dissolved in DMSO just before use.
Hydroxylamine (1%)	Hydroxylamine solution (wt. 50%) is 50-fold-diluted with ultrapure water. The solution can be stored at 25°C for two weeks.
CaCl_2_ (10 mM)	The solution can be stored at 25°C for three months.
Ammonium bicarbonate (50 mM)	The solution can be stored at 4°C for three months.

**CRITICAL:** To avoid inhalation of TFA and PMSF, perform all operations with these reagents in a certified chemical fume hood while wearing appropriate personal protective equipment, such as gloves and safety goggles.

***Alternatives:*** 3-[4-(2-Hydroxyethyl)-1-piperazinyl]propanesulfonic acid (EPPS), rather than HEPES, may be more suitable for buffering at pH 8.5 (Navarrete-Perea et al., 2018).

## Step-by-step method details

### Preparation of conditioned media

**Timing: 2–3 days**

For the identification of shedding substrates, it is preferable to investigate the proteins secreted into the cell culture media, rather than those remaining in the membrane, because the secretome is much less complex than the membrane proteome. Here, we prepare conditioned media of A431 cells treated with PMA and exposed to DMSO or BB-94 in order to quantitatively identify metalloprotease substrates that are downregulated by BB-94 treatment.

1. A431 cells are maintained in Dulbecco's modified Eagle’s medium (DMEM) supplemented with 10% fetal bovine serum, 100 U/mL penicillin and 100 μg/mL streptomycin. Seed 5–10 million A431 cells onto each of eighteen 15 cm dishes (3 dishes × 3 replicates × 2 conditions (DMSO or BB-94) = 18 dishes) on the day before media conditioning. Culture media from three dishes are merged as a replicate later.

***Note:*** The number of cells that should be seeded depends on the cell type. In our protocol, the media conditioning is performed with 90% confluent cells. We prepare more culture media than needed (in total 30 mL from three 15 cm dishes in this workflow) in order to ensure the availability of a sufficient amount of protein. The protein yields from the conditioned media of 10 human cell lines were reported in our previous paper (Tsumagari et al., 2021).

2. Wash cells three times with 20 mL of PBS(+).

***Note:*** Repetitive cell washing is important to avoid serum contamination. However, some cell lines, *e.g.*, HEK293T cells, easily become detached from the dish during cell washing. To minimize cell detachment, use PBS including Ca^2+^ and Mg^2+^ (PBS(+)), serum-free medium, or Hanks' balanced salt solution (HBSS) including Ca^2+^ and Mg^2+^ (HBSS(+)).

3. For inhibitor pretreatment, incubate cells with 10 mL of fresh serum-free medium including 10 μM BB-94 or DMSO at 37°C in a humidified incubator with 5% CO_2_ for 1 h. Note that the inhibitor pretreatment is required to distribute this drug into cells completely before activation of sheddases with PMA.
4. For media conditioning, remove the media for inhibitor pretreatment (step 3) and incubate the cells with 10 mL of fresh serum-free medium including 1 μM PMA and 10 μM BB-94 or DMSO at 37°C in a humidified incubator with 5% CO_2_ for 1 h.

***Note:*** HBSS(+) can be used instead of serum-free medium. For media conditioning, we employ as small a solution volume as possible to reduce the time required for concentration (step 8).

***Note:*** When using PMA, a shedding activator, we employ a short incubation time of 1 h. If such a reagent is not used, we recommend a longer incubation time such as for 16 h in the presence of inhibitor or DMSO to obtain a sufficient protein amount for the experiment (at least 20 μg: 10 μg each for N- and C-terminal peptide enrichment).

5. Collect the culture supernatant as conditioned media on ice. The supernatants of three dishes are pooled to make a biological replicate (total 30 mL).
6. Centrifuge the collected conditioned media at 3,000 × *g* for 30 min at 4°C to remove cell debris and transfer the supernatant to a fresh tube.
7. Add 0.3 mL of 200 mM EDTA-NaOH, 0.3 mL of 200 mM EGTA-NaOH, 0.3 mL of 100 mM PMSF, and 30 μL of 100x protease inhibitor cocktail (0.1% at final concentration) to 30 mL of conditioned medium.

**CRITICAL:** To avoid inhalation of PMSF, perform all operations with PMSF in a certified chemical fume hood while wearing appropriate personal protective equipment, such as gloves and safety goggles.

**Pause point:** Samples can be stored at –80°C for several weeks until use.

### Protein purification, reduction, and alkylation

**Timing: 8 h**

8. Transfer the conditioned medium to an Amicon Ultra centrifugal filter and centrifuge at 3,000 × *g* at 4°C to reduce the sample volume to 200 μL.

***Alternatives:*** Centrifugal filters from other manufacturers can be used, as long as they can reduce the sample volume to ∼200 μL. Follow the manufacturer’s instructions.

***Note:*** If the sample volume is larger than the capacity of the centrifugal filter, load and enrich as much sample as possible, and then separately load the remaining sample. The time needed for reducing the sample volume to 200 μL is likely to differ depending on the samples. In our experiments, it takes 4–7 h.

***Note:*** The sample volume should be reduced in order to perform protein extraction in a single microtube according to the following procedures. If the concentrated sample volume becomes less than 200 μL, add flow-through solution to adjust the volume.

9. Protein purification using methanol and chloroform. Transfer the sample from the Amicon Ultra centrifugal tube to a 2 mL microtube.
  a. Add 600 μL of methanol (3 times the sample volume), and mix vigorously for 1 min.
  b. Add 150 μL of chloroform (0.75 times the sample volume), and mix vigorously for 1 min.
  c. Add 450 μL of ultrapure water (2.25 times the sample volume), and mix vigorously for 1 min.
  d. Centrifuge at 20,000 × *g* for 5 min at 25°C .
  e. Remove the upper phase.
  f. Gently add 450 μL of methanol (2.25 times the sample volume), and carefully mix with the lower layer by gently swinging the tube.
    **CRITICAL:** Do not use vortex mixing at step 9f. If you see a white disc of protein (Figure 3), keep it as intact as possible. If the protein amount is very low, vigorous mixing could reduce the protein yield.
    **Figure 3:**
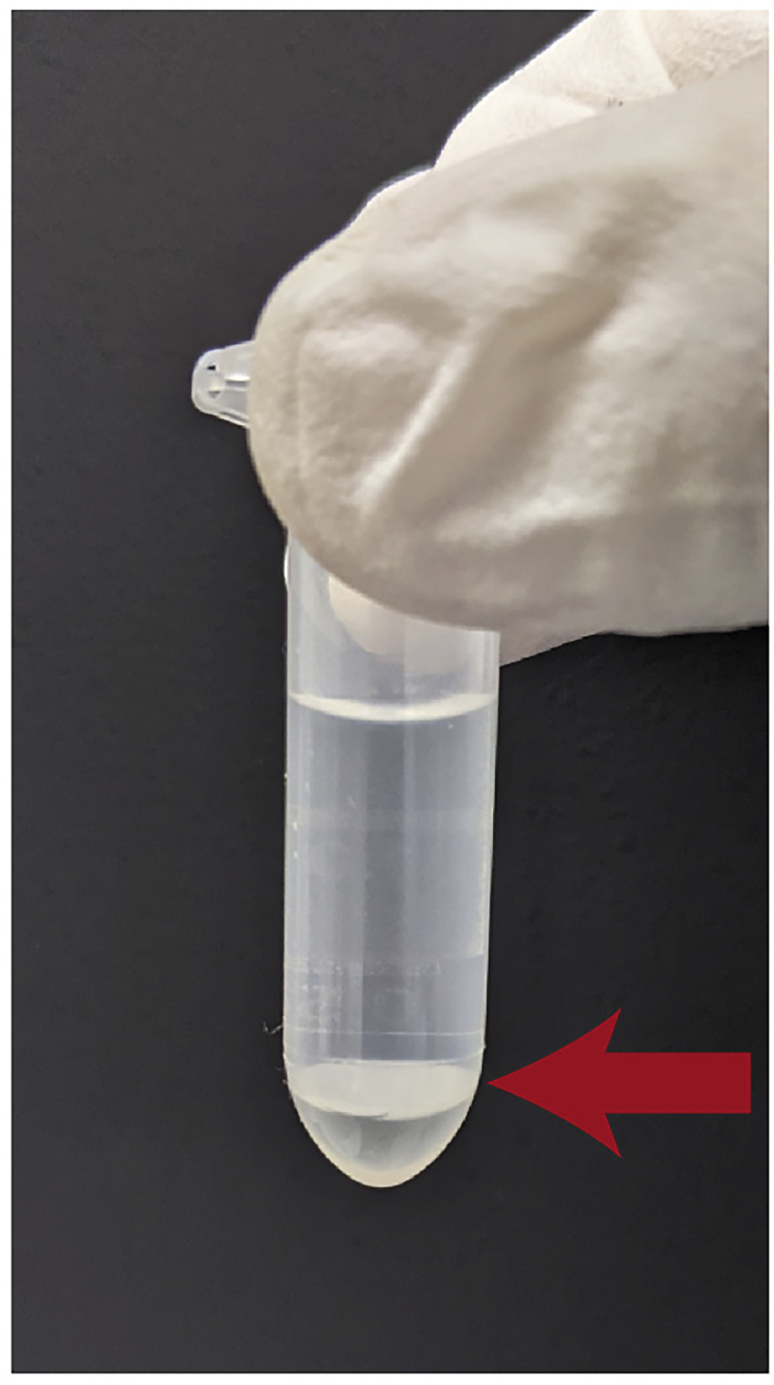
Example of protein purification with methanol-chloroform The white disc between the methanol and chloroform is a protein layer.

**Figure 4:**
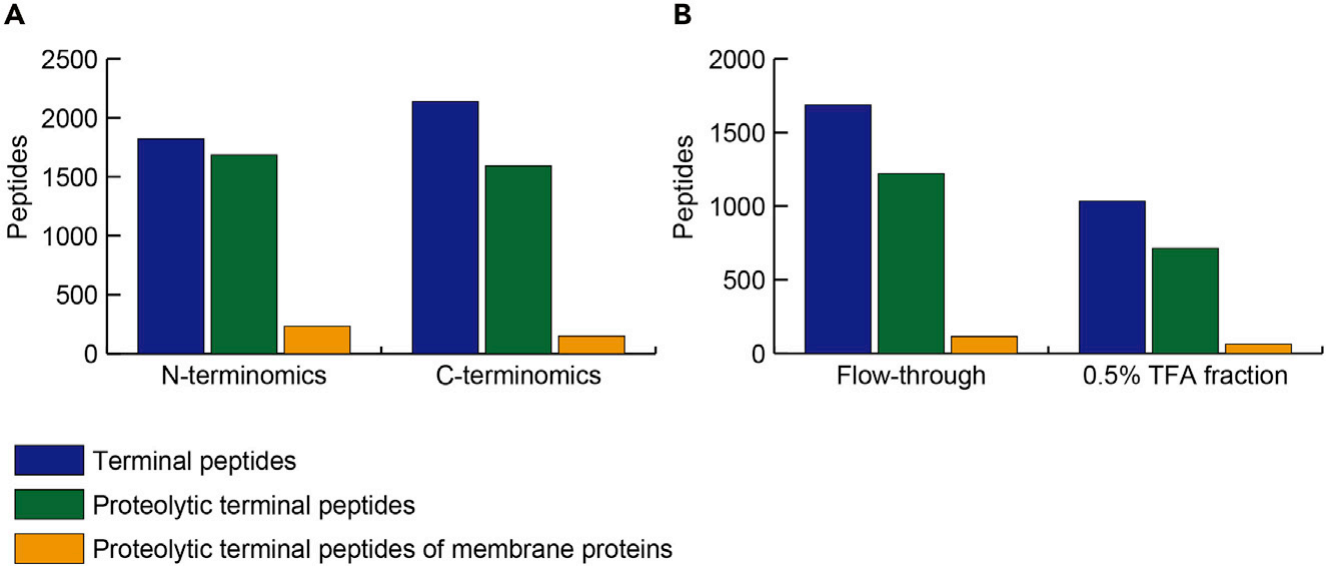
Number of identified peptides (A) The numbers of identified terminal peptides, proteolytic terminal peptides, and proteolytic terminal peptides in N- and C-terminal peptides are shown. Peptide uniqueness is based on sequence and modification. (B) The number of identified peptides in each fraction is shown for C-terminomics.
  g. Centrifuge at 20,000 × *g* for 5 min at 25°C.
  h. Remove the supernatant, and dry the pellet by leaving the tube tab open for 2 min.

10. Dissolve the protein pellet in 200 μL of PTS buffer.

***Note:*** If the protein pellet is hard to dissolve, sonication is effective.

11. Measure the protein concentration by BCA assay. The expected amount of protein yield is described in the Expected Outcomes section.

***Alternatives:*** Other protein quantification methods, such as Bradford assay, can be used.

12. Add 20 μL (1/10 volume) of 100 mM TCEP and 20 μL (1/10 volume) of 400 mM CAA, and mix the solution using a vortex mixer. Incubate at 95°C for 5 min.
13. Calculate the protein concentration of the sample considering the volume of added TCEP and CAA.

**Pause point:** The samples can be stored at –80°C for several weeks until use.

### Protein digestion for N-terminomics (LysargiNase digestion)

**Timing: 2 days**

Digestion is performed based on the reported phase-transfer surfactant (PTS) method (Chang et al., 2021; Masuda et al., 2009). PTSs, such as SDC and SLS, increase the solubility of hydrophobic proteins, and can be removed from acidified solution containing peptides by adding ethyl acetate. PTSs are predominantly transferred to the organic phase, while the digested peptides remain in the aqueous phase under the acidic condition. Importantly, peptides for N-terminal peptide enrichment should be prepared with LysargiNase, which cleaves proteins at the N-terminal side of Lys and Arg. In LysargiNase digests, non-terminal peptides have sequences starting with Lys or Arg, while N-terminal peptides have neither Lys nor Arg. Thus, N-terminal peptides can be isolated by means of SCX (steps 44–55) due to their weaker retention. In reported work (Chang et al., 2021; Tsumagari et al., 2021), we used TrypN, which is an alternative to LysargiNase purchased from Protifi (Cat#K-20), but this is no longer manufactured. Here, we set 10 μg peptides as input for one experiment.

14. Take 10 μg of protein sample dissolved in PTS buffer (from step 13).
15. Dilute 10-fold with 10 mM CaCl_2_.
16. Add 0.2 μg of LysargiNase (50:1, w/w).
17. Incubate for 16 h at 37°C with agitation at 700 rpm.
18. Add an equal volume of ethyl acetate and 10% by volume of 10% TFA (0.5% final concentration).
19. Vigorously mix with a vortex mixer for 2 min.
20. Centrifuge at 15,700–20,000 × *g* for 2 min at 25°C.
21. Carefully remove the upper phase (organic phase), which includes the surfactants.

***Note:*** You might see a white disc between the aqueous phase and the organic phase (similar to the disc observed in protein purification with methanol-chloroform, step 9; Figure 3). This does not affect the following analyses. Keep it intact while removing the organic phase.

22. Dry the peptide sample using a SpeedVac to remove remaining ethyl acetate.
23. Purify the peptide by using a reversed-phase StageTip as described later (steps 36–43).

**Pause point:** The peptide-loaded and washed StageTips can be stably stored at −20°C.

### Protein digestion for C-terminomics (trypsin/LysC digestion)

**Timing: 2 days**

Digestion is performed based on the PTS method (Chang et al., 2021; Masuda et al., 2009), as well as LysargiNase digestion (steps 14–23). Note that trypsin and LysC, instead of LysargiNase, should be used. Proteins are cleaved at C-terminal side of Lys and Arg in trypsin/LysC digestion. The non-terminal peptides have sequences ending with Lys or Arg, while C-terminal peptides have neither Lys nor Arg. Thus, C-terminal peptides can be isolated by means of SCX (steps 64–76) due to their weaker retention.

24. Take 10 μg of protein sample dissolved in PTS buffer (from step 13).
25. Dilute 5-fold with 50 mM ammonium bicarbonate.
26. Add 0.2 μg of LysC (50:1, w/w).
27. Incubate at 37°C for 3 h with agitation at 700 rpm.
28. Add 0.2 μg of trypsin (50:1, w/w).

***Note:*** 100:1 to 20:1 (protein:trypsin, w/w) can be used according to the manufacturer’s protocol.

29. Incubate 16 h at 37°C with agitation at 700 rpm.
30. Add an equal volume of ethyl acetate and 10% volume of 10% TFA (0.5% final concentration).
31. Vigorously mix with vortex for 2 min.
32. Centrifuge at 15,700–20,000 × *g* for 2 min at 25°C.
33. Carefully remove the upper phase (organic phase), which includes the surfactants.

***Note:*** You might see a white disc between the aqueous phase and the organic phase (similar to the disc observed in protein purification with methanol-chloroform, step 9; Figure 3). This does not affect the following analyses. Keep it intact during removal of the organic phase.

34. Dry the peptide sample using a SpeedVac to remove remaining ethyl acetate.
35. Purify the peptide on a reversed-phase StageTip as described later (steps 36–43).

**Pause point:** The peptide-loaded and washed StageTips can be stably stored at −20°C.

### Peptide purification

**Timing: 1 h**

In order to remove interfering chemicals, such as salts, peptides are purified by reversed-phase chromatography. Here, we use StageTips packed with double 16-gauge SDB-XC membranes. This peptide purification process appears 4 times in this workflow (N1, N2, C1, and C2; see Figure 1). In order to distinguish these repeated steps, the respective peptide purification steps are referred to as, for example, “Peptide purification (N1)” or “step 43 (N1)”.

**CRITICAL:** In peptide purification of TMT-labeled trypsin/LysC digests (C2; Figure 1), the peptide amount is in total 60 μg (10 μg × 6 samples are mixed at step 61). Thus, divide the digests into three parts and purify them using three StageTips, considering the SDB-XC membrane capacity. Otherwise, use single StageTip per sample.

36. Dissolve the peptide sample (from steps 23, 35, or 63) in 20–40 μL of buffer A.

***Note:*** If the peptide pellet is hard to dissolve (particularly peptides just after digestion), add 2 M guanidine chloride to buffer A in this step. Chaotropic agents such as guanidine chloride facilitates dissolution. Sonication for 2–5 min is also effective.

37. Prepare SDB-XC StageTips, and set 2 mL microtubes as receivers.
38. *Conditioning.* Wet the membranes by passing 50 μL of buffer B through the StageTip.
39. *Equilibration.* Add 50 μL of buffer A to the StageTip just before buffer B has completely left the tip, and pass buffer A through the tip. Stop before buffer A has completely left the tip.
40. *Sample loading.* Load the peptide sample (from step 36) onto the tip, and pass the sample solvent through the tip.
41. *Wash.* Add 50 μL of buffer A just before the sample has completely left the tip, and pass buffer A through the tip.

**Pause point:** The peptide-loaded and washed StageTips can be stably stored at −20°C.

**CRITICAL:** Change the receiver tube to a new 1.5 mL microtube to collect the eluate at the next step.

42. *Elution.* Apply 50 μL of buffer B, and collect the eluate.
43. Dry the eluate in a SpeedVac.

### N-terminal peptide enrichment

**Timing: 2 h**

The N-terminal peptides in LysargiNase-digests can be separated from the non-terminal peptides on SCX due to their weaker retention by SCX at low pH. The principle was described in detail previously (Chang et al., 2021).

**CRITICAL:** Isobaric labeling such as TMT alters the charge distribution of peptides and affects the enrichment efficiency. The TMT-labeling step should be performed after N-terminal peptide enrichment, which is different from the case of C-terminal peptide enrichment (Figure 1).

44. Dissolve the purified LysargiNase digests (from step 43 (N1, Figure 1)) of a 10 μg protein sample in 50 μL of N-loading buffer.
45. Prepare a StageTip with double 16-gauge SCX membranes, and set it on a 2 mL microtube as a receiver tube.
46. *Conditioning.* Wet the membranes by passing 50 μL of methanol through the StageTip.
47. Add 50 μL of buffer B to the StageTip just before the methanol has completely left the tip, and pass buffer B through the tip.
48. *Activation.* Add 100 μL of SCX-activation buffer to the StageTip just before buffer B has completely left the tip, and pass SCX-activation buffer through the tip.
49. *Equilibration.* Add 150 μL of N-loading buffer to the StageTip just before the SCX-activation buffer has completely left the tip, and pass N-loading buffer through the tip.
50. Again, add 150 μL of N-loading buffer to the StageTip just before the previously applied N-loading buffer has left the tip, and pass N-loading buffer through the tip. Stop before the N-loading buffer has completely left the tip.

**CRITICAL:** Change the receiver tube to a new 1.5 mL microtube to collect the flow-through fraction of steps 51–52 in the following sample loading step and wash step.

51. *Sample loading.* Load the sample dissolved in N-loading buffer (from step 44) onto the tip, and pass the sample through the tip.
52. *Wash.* Add 50 μL of N-loading buffer just before the sample has completely left the tip, and pass the N-loading buffer through the tip.
53. Collect the solution (flow-through of steps 51–52) in the receiver tube as an N-terminal peptide sample.
54. Dry the obtained N-terminal peptides in a SpeedVac.
55. Label the enriched peptides with TMT according to the following steps 56–63.

**Pause point:** The evaporated peptides can be stored at –80°C for several weeks until use.

### TMT labeling

**Timing: 2 h**

Samples are multiplexed with TMT reagents (Table 1). TMT-labeling is particularly important in the C-terminal peptide enrichment step, since the additional positive charges on the peptides facilitate separation of C-terminal peptides from non-terminal peptides. If sample multiplexing is not employed, we recommend the use of TMT-zero reagent (Cat#90067, Thermo Fisher Scientific), which is much cheaper than TMT reagents labeled with stable isotopes. The N-terminal peptides should be TMT-labeled after N-terminal peptide enrichment, because TMT-labeling affects the enrichment efficiency in N-terminal peptide enrichment (steps 36–43, Figure 1).

56. Dissolve the peptides (from step 43 (C1) or 55) in 5 μL of 200 mM HEPES-NaOH (pH 8.5).

***Alternatives:*** 3-[4-(2-Hydroxyethyl)-1-piperazinyl]propanesulfonic acid (EPPS), rather than HEPES, may be more suitable for buffering at pH 8.5 (Navarrete-Perea et al., 2018).

57. Mix the peptide solution with 0.1 mg of TMT reagent dissolved in 5 μL of acetonitrile in a vortex mixer.
58. Incubate the solution at 25°C with agitation at 700 rpm.
59. Add 5 μL of 1% hydroxylamine for quenching excess reagents.
60. Add 10 μL of 10% TFA to stop the reaction.
61. Mix the samples (TMT 6-plex).
62. Dry the sample in a SpeedVac.
63. Purify the peptide using a reversed-phase StageTip as described already (steps 36–43). See the “CRITICAL” description regarding the peptide purification (C2) process.

**Table 1:** Example of sample multiplexing

TMT channel	N-Terminal peptides	C-terminal peptides
126	DMSO rep.1	DMSO rep.1
127N	DMSO rep.2	DMSO rep.2
128C	DMSO rep.3	DMSO rep.3
129N	BB-94 rep.1	BB-94 rep.1
130C	BB-94 rep.2	BB-94 rep.2
131	BB-94 rep.3	BB-94 rep.3

### C-terminal peptide enrichment

**Timing: 2 h**

In our protocol for C-terminal peptide enrichment, the C-terminal peptides in trypsin/LysC-digests can be separated from the non-terminal peptides by SCX due to their weaker retention on SCX at low pH. TMT labeling introduces additional positive charges on the peptides, facilitating the separation of C-terminal peptides from non-terminal peptides. Thus, even if you do not perform a multiplexed experiment, label the peptides with TMT-zero reagent. The 6-plexed TMT sample is subjected to C-terminal peptide enrichment using three SCX-StageTips (Figure 1).

64. Dissolve 60 μg of purified TMT-labeled peptides (from step 43 (C2)) in 150 μL of C-loading buffer.
65. Prepare three SCX-StageTips, and set them on 2 mL microtubes as receiver tubes.
66. *Conditioning.* Wet the SCX-membranes by passing 50 μL of methanol through the StageTip.
67. Add 50 μL of buffer B to the StageTip just before the methanol has completely left the tip, and pass buffer B through the tip.
68. *Activation.* Add 100 μL of SCX-activation buffer to the StageTip just before buffer B has completely left the tip, and pass SCX-activation buffer through the tip.
69. *Equilibration.* Add 150 μL of C-loading buffer to the StageTip just before the SCX-activation buffer has completely left the tip, and pass C-loading buffer through the tip.
70. Again, add 150 μL of C-loading buffer to the StageTip just before the previously applied C-loading buffer has completely left the tip, and pass C-loading buffer through the tip. Stop before the C-loading buffer has completely left the tip.

**CRITICAL:** Change the receiver tube to a new 1.5 mL microtube to collect the flow-through fraction in the following sample loading step and wash step.

71. *Sample loading.* Load 50 μL (one-third) of sample solution dissolved in C-loading buffer (from step 64) onto the tip.
72. *Wash.* Add 50 μL of C-loading buffer just before the sample has completely left the tip, and pass the C-loading buffer through the tip.
73. Collect the solution (flow-through of steps 71–72) in the receiver tube as the “flow-through fraction” of C-terminal peptides.

**CRITICAL:** Change the receiver tube to a new 1.5 mL microtube to collect the peptides eluted by C-0.5% TFA buffer in the next step.

74. Add 50 μL of C-0.5% TFA buffer to the StageTip just before the C-loading buffer has completely left the tip, and pass C-0.5% TFA buffer through the tip.
75. Collect the eluate as the “0.5% TFA fraction” of C-terminal peptides.
76. Dry the obtained C-terminal peptides in a SpeedVac.

***Note:*** The evaporated peptide sample can be subjected to nanoLC/MS/MS analysis.

### Nano-scale liquid chromatography/tandem mass spectrometry

**Timing: 24 h**

For N-terminal peptide analysis, one-third of the peptides is injected, and triplicate analyses are carried out (3 LC/MS/MS runs). For C-terminal peptide analysis, three sets of flow-through and 0.5% TFA fractions are subjected to single-shot analysis, respectively (6 LC/MS/MS runs) (Figure 1).

Here, we describe an example of nanoLC/MS/MS analysis, which we employed in our reported work (Tsumagari et al., 2021). The nanoLC/MS/MS system comprises an UltiMate 3000RSLCnano pump (Thermo Fisher Scientific) and an Orbitrap Fusion Lumos tribrid mass spectrometer (Thermo Fisher Scientific). Peptides are injected by an HTC-PAL autosampler (CTC Analytics), loaded onto a 15 cm fused-silica emitter packed with ReproSil-Pur C18-AQ (3 μm; Dr. Maisch), and separated by a linear gradient, that is, 5% B for 1 min, 5–15% B in 4 min, 15–40% B in 100 min, 40–99% B in 5 min, and 99% B for 10 min (Solvent A, 0.5% acetic acid; solvent B, 0.5% acetic acid in 80% ACN) at the flow rate of 500 nL/min. The ionization voltage is set to 2,400 V. All MS1 spectra are acquired over the range of 375–1500 *m/z* in the Orbitrap analyzer (resolution = 120,000, maximum injection time = 50 ms, automatic gain control = 4e5). For the subsequent MS/MS analysis, precursor ions are selected and isolated in top-speed mode (cycle time = 3 s, isolation window = 1.4 *m/z*), activated by higher-energy collisional dissociation (HCD; normalized collision energy = 38), and separated and detected in the Orbitrap analyzer (resolution = 50,000, maximum injection time = 105 ms, automatic gain control = 1e5). The dynamic exclusion time is set to 30 s.

***Note:*** In order to increase the accuracy of peptide identification by database searching, we recommend using the high-resolution Orbitrap analyzer for both MS1 and MS2 acquisition.

### NanoLC/MS/MS raw data processing

**Timing: 6 h**

We here describe an example of nanoLC/MS/MS raw data processing using MaxQuant (v.1.6.7.0), which is a free and widely used suite including the Andromeda search engine (Cox and Mann, 2008; Cox et al., 2011; Tyanova et al., 2016a). Database search is implemented against the UniProtKB/SwissProt (https://www.uniprot.org/) human database, concatenated with commonly observed contaminant protein sequences set in MaxQuant. The following parameters are applied: two analysis groups were made in MaxQuant, enabling one combined analysis for LysargiNase with N-terminal free semi-specificity (N-terminal peptides) and trypsin/P with C-terminal free semi-specificity (C-terminal peptides); analysis type is set to 10-plexed TMT quantification at the MS2 level; minimal peptide length is set to 7 amino acids; Cysteine carbamidomethylation is set as a fixed modification, while methionine oxidation and acetylation on the protein N terminus are allowed as variable modifications; false discovery rate is set to 1% at the peptide-spectrum match and protein level, respectively. Details of the procedure for usage of MaxQuant have been described previously (Tyanova et al., 2016a).

***Note:*** The time needed for the data processing is strongly dependent on the performance of the PC running the software.

## Expected outcomes

The protein yield (step 11) is strongly dependent on the cell type. In our previous work, the yields ranged roughly from 20 to 300 μg per replicate among 10 human cancer cell lines. This variability is presumably due to the differences of intrinsic characteristics such as expression of sheddases and activity of protein secretion. In the case of A431 cells, roughly 40–100 μg of proteins is obtained. Note that drug treatment can also affect the protein yields; BB-94-treated samples are likely to give lower protein yields than controls, as was observed for most of the cell lines we have investigated (Tsumagari et al., 2021).

The number of identified terminal peptides from A431 cells is shown in Figure 4. The expected enrichment efficiency in terminal peptide enrichment is as follows: 77% in N-terminal peptide enrichment; roughly 38% in flow-through fraction and roughly 13% in 0.5% TFA-fraction in C-terminal peptide enrichment (calculated as the number of terminal peptides divided by the number of total peptides). In C-terminomics, two fractions, the flow-through and the 0.5% TFA-eluted fraction, are analyzed, which allows identification and quantification of C-terminal peptides on a comparable scale to the N-terminal peptide counterpart, even though the enrichment efficiency is inferior to that of N-terminal peptide enrichment. Note that the enrichment efficiency is lower when calculated in term of the number of peptides, because the highly sensitive nanoLC/MS/MS system identifies even small amounts of non-terminal peptides present as contaminants. In our previous report, the estimated enrichment efficiency were roughly 82% at peptide count level and roughly 98% at peak area level (Chang et al., 2021). The enrichment efficiency is likely to be different depending on the sample used (Tsumagari et al., 2021).

Examples of the identified terminal peptides from A431 cells are shown in Table 2. The list processing procedure is described in the following Quantification and Statistical Analysis section. Note that the number of identified peptides is strongly dependent on the cell type (Tsumagari et al., 2021).

**Table 2:** Examples of BB-94-downregulated proteolytic peptides derived from membrane proteins

Gene names	UniProt accession	Identified peptide sequence	Cleavage window^(a)^	P1^(b)^	-LOG p value	Log_2_ (BB-94/DMSO)	N-Terminal or C-terminal peptide
CANX	P27824	AADGAAEPGVVGQ	VVGQ↓MIEA	473	1.30	-2.53	C
ALCAM	Q13740	ADIQMPF	QMPF↓TCSV	219	3.77	-2.44	C
PTK7	Q13308	SEGPGSPPPY	VPEE↓SEGP	690	4.04	-2.29	N
SDC1	P18827	NQSPVDQGATGA	ATGA↓SQGL	243	3.79	-2.14	C
MRC2	Q9UBG0	AEQSSFSP	SFSP↓SALP	1406	3.58	-2.11	C
PVRL4	Q96NY8	DSQVTVDVLDPQE	DPQE↓DSGK	338	3.92	-2.05	C
PTPRF	P10586	NGVITQY	ITQY↓SVAY	643	3.75	-1.99	C
LY6D	Q14210	VSSGTSSTQCCQEDLCNE	LQGQ↓VSSG	77	3.08	-1.97	N
HLA-B	P01889	MYGCDVGPDG	TLQS↓MYGC	122	4.12	-1.96	N
CDH1	P12830	VSVCDCEGAAGVC	TTLE↓VSVC	683	2.66	-1.85	N
HLA-C	P01889	ISVGYVDDTQFV	EPRF↓ISVG	47	3.34	-1.83	N
ALCAM	Q13740	QIGDALPVSC	PVSC↓TISA	355	3.29	-1.82	C
PTPRU	Q92729	EPGGQDCFPVPLTFEAALA	RLRR↓EPGG	640	3.37	-1.81	N
LMAN2	Q12907	IEPSVNF	SVNF↓LKSP	299	5.76	-1.72	C
ITM2B	Q9Y287	LYQTIEENI	APAA↓LYQT	111	3.62	-1.72	N
PTK7	Q13308	GPPIILEA	ILEA↓TLHL	316	3.97	-1.70	C
HLA-C	P01889	WTAADTAAQITQ	DLRS↓WTAA	157	2.73	-1.69	N
APP	P05067	EQNYSDDVLAN	VLAN↓MISE	580	3.12	-1.69	C
EFNB1	P98172	SGGSSGDPDGFFNS	GPGA↓SGGS	223	3.28	-1.66	N
CDH3	P22223	LTVTDLDAPNSPAW	EVQR↓LTVT	349	3.02	-1.64	N

Top 10 downregulated N- and C-proteolytic peptides are listed in ascending order of the ratio. (a) The amino acids flanking the identified cleavage sites (±4) are shown. An arrow (↓) indicates the cleavage site. (b) The number of the P1 position (1 amino acid upstream of the cleavage site) is shown.

## Quantification and statistical analysis

**Figure 5:**
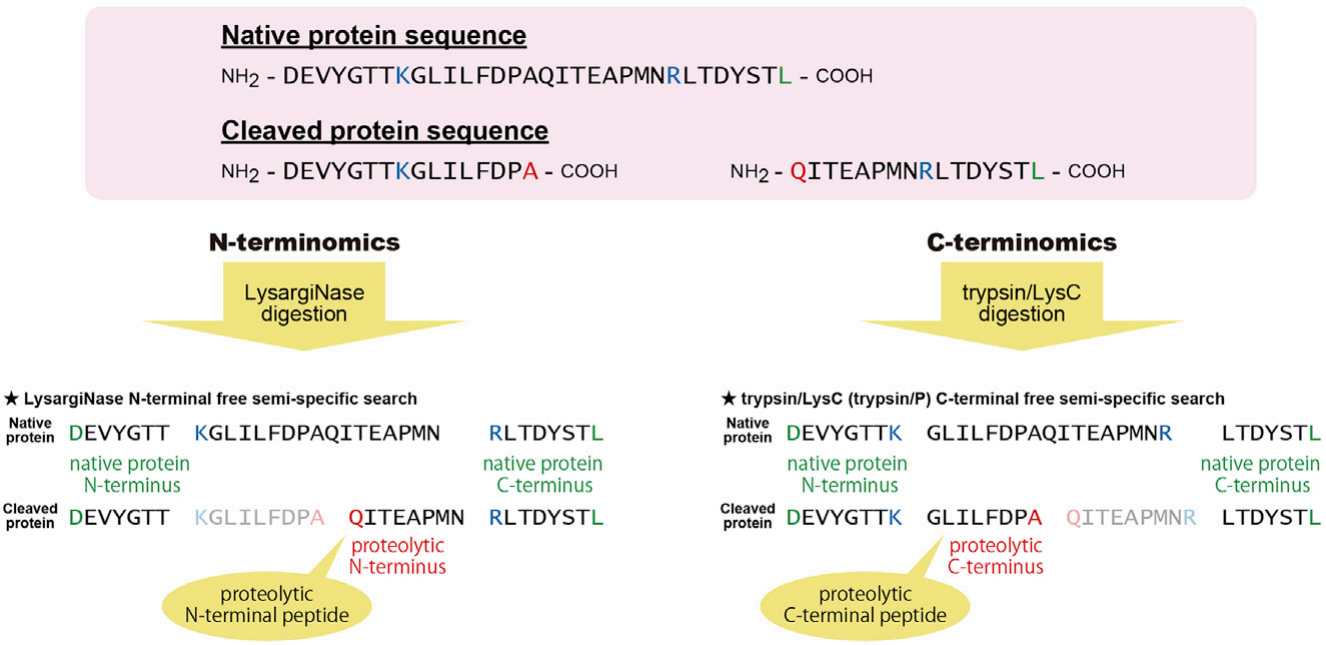
Example of terminal peptide and cleavage site identification by semi-specific searches In standard shotgun proteomics, only peptides whose N- and C-termini are both in accordance with the specificity of the used enzyme are considered. The N terminus of native protein N-terminal peptide (highlighted in green) does not match to the specificity of LysargiNase (cleavage at N-terminal side of K/R, highlighted in blue), but can be identified in the conventional LysargiNase-specific search. In N-terminal free semi-specific search, peptides whose N terminus does not match the specificity of LysargiNase (highlighted in red) can be additionally identified. Similarly, the C terminus of native protein C-terminal peptide (highlighted in green) does not match the specificity of trypsin/LysC (trypsin/P; cleavage at the C-terminal side of K/R, highlighted in blue), but can be identified in the conventional trypsin/P-specific search. In C-terminal free semi-specific search, peptides whose C terminus does not match the specificity of trypsin/LysC (highlighted in red) can be additionally identified.

We use the “Peptides.txt” file given by MaxQuant for analysis. We exclude peptides with missed cleavages from further analyses in order to simplify the relationship between the cleavage sites and the corresponding peptides. In N-terminomics, in which LysargiNase is used for digestion, the non-terminal peptides have sequences starting with K or R, while N-terminal peptides have neither K nor R. Similarly, in C-terminomics, in which trypsin and LysC are used for digestion, the non-terminal peptides have sequences ending with K or R, while C-terminal peptides have neither K nor R. Based on these facts, we extract N- and C-terminal peptides, respectively (Figure 5). In order to find terminal peptides containing cleavage sites targeted by intrinsic proteases, we extract terminal peptides with a terminus not cleaved by the spiked digestive enzymes. Such terminal peptides would be derived from intrinsic proteolytic events, such as ectodomain shedding. We refer to such terminal peptides as “proteolytic peptides” and the presented termini as “proteolytic termini” (Figure 5).

**Figure 6:**
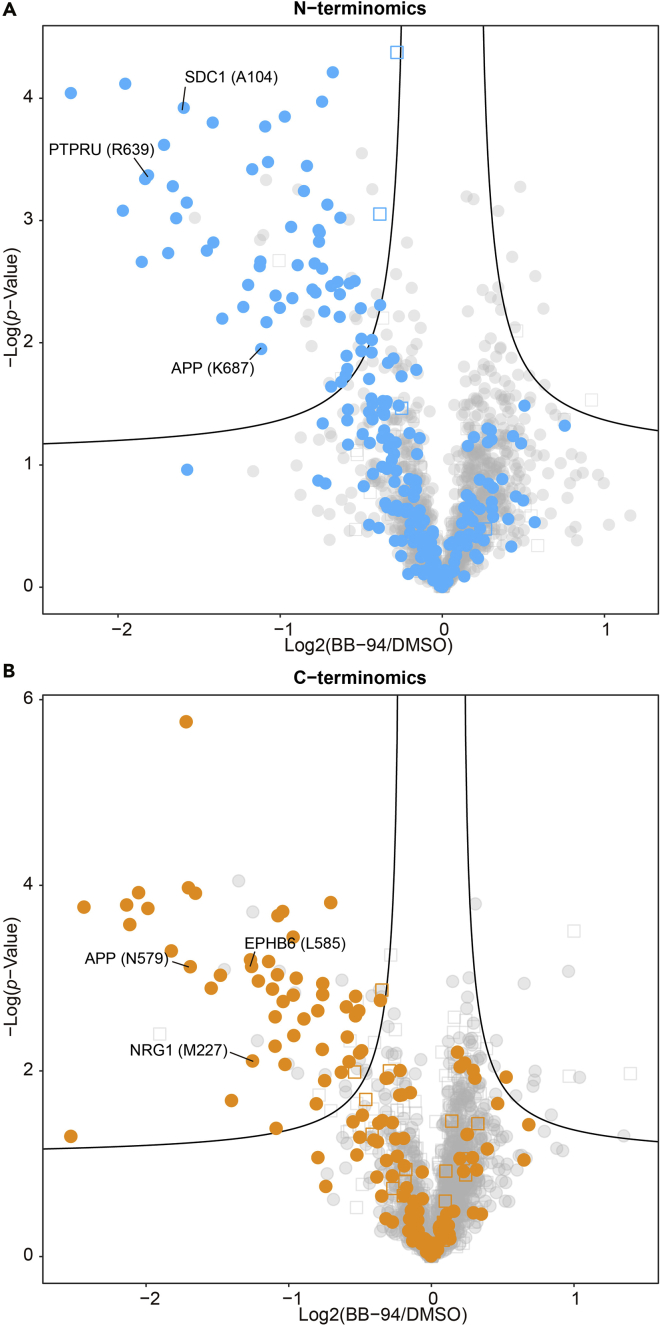
Volcano plots Volcano plots are created for (A) N-terminal peptides and (B) C-terminal peptides using Perseus with default parameters (truncation at the false discovery rate of 0.05 and an artificial within groups variance [S_0_] of 0.1). Native protein termini are shown with open squares, while proteolytic protein termini are shown with closed circles. Membrane proteins are highlighted in color.

Here, we introduce a simple normalization strategy that does not need any programing skill: the medians of the log_2_-converted intensities are adjusted to 0 by subtracting the median values in the respective TMT-channels (median normalization). Membrane proteins are defined with UniProt keywords “GPI-anchor” and “transmembrane”. UniProt Keywords annotation and following statistical analysis are performed by using Perseus software, which is freely available (Tyanova et al., 2016b). We create volcano plots, which have log_2_ ratios on the x-axis and -log_10_ *p-value*s on the y-axis, with the default truncation parameters (randomization = 250, FDR <0.05, S_0_ = 0.1) (Figure 6). The A431 N-terminomics and C-terminomics datasets afforded in total 109 cleavage sites on membrane proteins as putative shedding substrates (Table 2).

## Limitations

First, this protocol can only be applied to cell types that can be maintained in serum-free media to prepare conditioned media. Second, termini that are not accessible to LysargiNase or LysC/trypsin, i.e., terminal peptides that are too short or too long, would not be captured by our methodology: for instance, short peptides, such as dipeptides and tripeptides, can be sequenced by database searches, but it is difficult to uniquely identify which protein they were digested from. In addition, long peptides with molecular weights greater than 3000 have *m/z* values greater than 1500 for doubly charged peptide ions, which is outside the scan range of LC/MS/MS. Third, terminal peptides with multiple histidine residues are not isolated because their positive charge is too large to enable separation from non-terminal peptides. Finally, it should be noted that we have not performed biochemical validation of the specificity of spiked proteases utilized for sample preparation. Thus, there is a possibility that identified semi-specific peptides may have been generated by digestion at unpredicted sites during sample preparation; however, this seems unlikely, as previous studies have demonstrated extremely high specificity, particularly for trypsin (Olsen et al., 2004; Wilson et al., 2020).

## Troubleshooting

### Problem 1

Low protein yield

### Potential solution

Protein yield is strongly dependent on the cell type (step 11). In our previous work, the protein yields obtained under the described conditions ranged roughly from 20 to 300 μg. If the yields are not sufficient for analysis, the scale of the culture should be increased. If shedding activator treatment is not used, extending the incubation time also increases the protein yield.

### Problem 2

Low digestion efficiency

### Potential solution

Too low a concentration of digestive enzyme (steps 16–17 or 26–29) can result in low digestion efficiency. Reduce the solution volume, or increase the amount of the digestion enzyme.

### Problem 3

High column pressure of StageTip (in all steps)

### Potential solution

The membrane disks were pressed into position too hard when the StageTip was manufactured (steps 37, 45, and 65). Use less force.

### Problem 4

High column pressure of StageTip (during sample loading)

### Potential solution

The sample contains small particles that are not completely dissolved in the solution and that clog the StageTip membrane surface. Split the sample on several StageTips and/or use StageTips membranes with larger diameter. Alternatively, sonication of the sample might facilitate dissolution of the particles.

### Problem 5

Low terminal peptide enrichment efficiency

### Potential solution

Salts should be completely removed by reversed-phase chromatography (peptide purification N1 or C2 (steps 36–43), Figure 1) prior to terminal peptide enrichment. Confirm that peptide purification is performed appropriately. Too high peptide concentration could lead to low efficiency. The loading speed of the solution through the membrane is also important. Keep the speed at approximately 10 μL/min.

## Resource availability

### Lead contact

Further information and requests for resources and reagents should be directed to and will be fulfilled by the lead contact, Yasushi Ishihama (yishiham@pharm.kyoto-u.ac.jp).

### Materials availability

This study did not generate new reagents.

### Data and code availability

The raw LC/MS/MS data analyzed in this study have been deposited with the ProteomeXchange Consortium (http://proteomecentral.proteomexchange.org) via the jPOST partner repository (https://jpostdb.org) with the data set identifier PXD021378 (JPST000632).
